# The Effect of Low-Flow and Normal-Flow Desflurane Anesthesia on the Frontal QRS-T Angle in Patients Undergoing Rhinoplasty Operation: A Randomized Prospective Study

**DOI:** 10.7759/cureus.28920

**Published:** 2022-09-08

**Authors:** Tugba Bingol Tanriverdi, Mehmet Tercan, Gulcin Patmano, Zulkif Tanriverdi, Ayse Güsun Halitoglu, Ahmet Kaya

**Affiliations:** 1 Department of Anesthesiology and Reanimation, University of Health Sciences, Mehmet Akif Inan Training and Research Hospital, Şanliurfa, TUR; 2 Department of Anesthesiology and Reanimation, University of Health Sciences, Kayseri Training and Research Hospital, Kayseri, TUR; 3 Department of Cardiology, Harran University, Faculty of Medicine, Şanliurfa, TUR; 4 Department of Anesthesiology and Reanimation, Mersin University, Faculty of Medicine, Mersin, TUR

**Keywords:** frontal qrs-t angle, myocardial repolarization, electrocardography, desflurane, low flow anesthesia

## Abstract

Introduction: Low-flow anesthesia (LFA) has gained more interest worldwide owing to its economic and ecological advantages compared to normal-flow anesthesia (NFA). Desflurane is one of the commonly used anesthetic agents for LFA, but it may prolong myocardial repolarization. Frontal QRS-T angle (f[QRS-T]a) is a novel marker of myocardial repolarization. To our knowledge, no study has compared the effect of LFA and NFA on f(QRS-T)a. In this study, we aimed to compare the effect of the LFA and NFA with desflurane on f(QRS-T)a in patients undergoing rhinoplasty operation.

Methods: A total of 80 patients undergoing rhinoplasty operations were included in this prospective study. The patients were randomized into two groups as follows: LFA (n = 40) and NFA (n = 40). The frontal QRS-T angle was calculated from the automatic report of the electrocardiography device (Nihon Kohden, Tokyo, Japan). It was recorded at the following time points: T1: preoperative (basal), T2: immediately after anesthesia induction, T3: immediately after endotracheal intubation, T4: 5 min after endotracheal intubation, T5: 15 min after endotracheal intubation, T6: 30 min after endotracheal intubation, T7: 60 min after endotracheal intubation, T8: end of the operation, T9: 15 min after the end of the operation.

Results: Baseline clinical characteristics and laboratory parameters were similar between the two groups. In the LFA group, f(QRS-T)a was significantly increased at only the T3 time point when compared to T1 (P = 0.003). However, in the NFA group, f(QRS-T)a was significantly increased at T3, T4, T5, T6, T7, T8, and T9 time points when compared to the T1 value (P < 0.05, for all). On the other hand, fQRS-Ta was significantly higher in the NFA group than in the LFA group at T4, T5, and T6 time points.

Conclusion: In our study, we have shown for the first time that NFA significantly increased the f(QRS-T)a, whereas LFA did not significantly increase the f(QRS-T)a except for immediately after the endotracheal intubation. It was also detected that f(QRS-T)a was significantly higher in the NFA group compared to that in the LFA group. Therefore, it can be concluded that LFA has more protective effects on myocardial repolarization than NFA.

## Introduction

Low-flow anesthesia (LFA) is an anesthesia technique and has recently gained more interest worldwide. Compared to normal-flow anesthesia (NFA) in which the flow rate of fresh gas to the breathing system is generally at 2 L/min, the gas flow rate is ≤1 L/min, and the re-inhaled fresh oxygen flow rate is at least 50% with a sufficient amount of volatile agent in LFA [1,2]. Previous studies reported many advantages of LFA. These include a reduced amount of volatile anesthetic agent, improved body temperature, less air pollution, and better protection of pulmonary function [2-4]. Therefore, LFA is physiologically more acceptable owing to these advantages.

Volatile anesthetic agents have been reported to prolong myocardial depolarization and repolarization with a direct effect [5-7]. Prolonged myocardial repolarization is associated with an increased risk of life-threatening arrhythmias. Myocardial repolarization is traditionally measured by QT and T peak to end (Tp-e) interval from surface electrocardiography (ECG) [8]. However, some technical difficulties in the measurement of QT and Tp-e intervals reduce their robustness and reproducibility [9]. Therefore, novel electrocardiographic markers of depolarization and repolarization have emerged recently. Frontal QRS-T angle (f[QRS-T]a) is a novel marker of myocardial depolarization and repolarization heterogeneity. It is defined as the absolute difference between myocardial depolarization (defined as the QRS axis in ECG) and repolarization (defined as the T axis in ECG). Because most of the ECG devices report these axes automatically, f[QRS-T]a can easily be measured from the 12-lead surface ECG [10].

Anesthesiologists tend to use LFA with desflurane and sevoflurane owing to their cost [11]. Desflurane is one of the commonly used volatile anesthetic agents in daily practice due to its less soluble structure and wide dose range. These properties make it an optimal agent for LFA [12]. Previous studies demonstrated that desflurane anesthesia prolonged myocardial repolarization [6,7,13,14]. However, the effect of low-flow and normal-flow desflurane anesthesia on myocardial repolarization has not been clearly demonstrated yet. In this study, we aimed to compare the effect of low-flow and normal-flow desflurane anesthesia on f(QRS-T)a in patients undergoing rhinoplasty operations.

## Materials and methods

A total of 80 patients undergoing rhinoplasty operation with the American Society of Anesthesiologists (ASA) status I or II were included in this randomized prospective study. Patients under the age of 18 and over the age of 65, those with hypertension, diabetes mellitus, heart failure, coronary artery disease, moderate/severe valvular disease, chronic renal or hepatic disease, complete/incomplete bundle branch block, anemia, electrolyte imbalance, and those taking drugs known to prolong myocardial repolarization were excluded from the study. Harran University Clinical Research Ethic Committee approved the study design (number: 03/10) and informed consent was obtained from all patients. Our study was also registered at the Australian New Zealand Clinical Trials Registry (number: ACTRN12621000408886).

Patients were taken to the operation room equipped with ASA monitoring, i.e ECG, heart rate (HR), mean arterial pressure (MAP), and oxygen saturation (SpO_2_). Anesthesia induction was performed with propofol (2 mg/kg) and fentanyl (1 µg/kg). Endotracheal intubation was performed after muscle relaxation with rocuronium 0.5 mg/kg. Patients were then connected to the mechanical ventilator. Anesthesia was maintained with desflurane (50% oxygen and 50% air mixture with desflurane, minimum alveolar concentration [MAC] value was targeted as +1). Patients were randomly allocated according to sealed opaque envelopes into two groups as LFA and NFA. LFA (n=40) was performed as the fresh gas flow rate was at 4-6 L/min for the first 6-8 minutes. After achieving MAC +1, the rate of fresh gas flow was reduced to 0.5 L/min. NFA (n=40) was performed as the fresh gas flow rate was at 4-6 L/min for the first 6-8 minutes. After achieving MAC +1, the rate of fresh gas flow was reduced to 2 L/min. During anesthesia maintenance, the target end-tidal CO_2_ value was 35-45 mmHg. Fifteen minutes before the end of the surgical operation, the vaporizer was turned off. In addition, 100% oxygen was performed at a 5-6 L/min rate for 3-5 min before extubating. The following time points were defined for the evaluation: T1: preoperative (basal), T2: immediately after anesthesia induction, T3: immediately after endotracheal intubation, T4: 5 min after intubation, T5: 15 min after intubation, T6: 30 min after intubation, T7: 60 min after intubation, T8: end of the operation, T9: 15 min after the end of the operation. Baseline characteristics, ASA status, duration of the procedure, laboratory parameters, HR, MAP, and electrocardiographic variables were recorded for all patients. 

**Figure 1:**
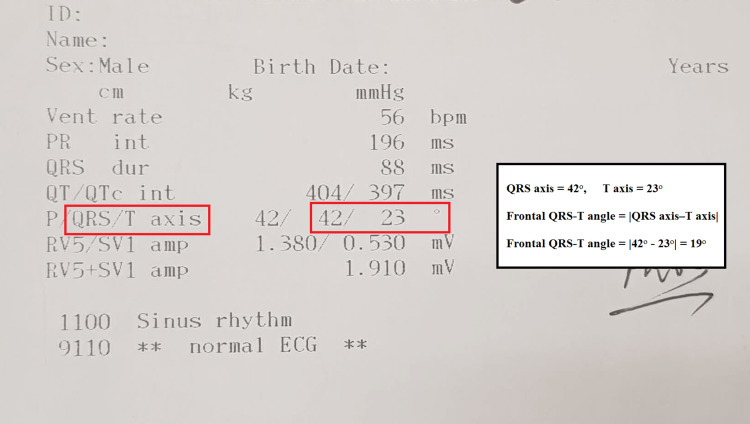
An example of the calculation of the frontal QRS-T angle from the automatic report of 12-lead electrocardiography.

Twelve-lead ECG was performed with a 25 mm/s speed, 10 mm/mV height, and 0.16-100 Hz. filter range (Nihon Kohden, Tokyo, Japan). The automatic report of the ECG was also printed. The frontal QRS axis and T axis were available in this automatic report of the ECG device. The frontal QRS-T angle was calculated from these axes as follows: the absolute difference between the QRS axis and T axis (frontal QRS-T angle = │QRS axis-T axis│). If this angle exceeded 180^o^, the calculation of the angle was again done by subtracting from 360^o^ [10,15,16]. An example of the calculation of f(QRS-T)a is demonstrated in Figure 1.

Statistical analyses were performed with SPSS 21.0 (IBM Corp, Armonk, NY). Kolmogorov-Smirnov test was used to evaluate the distribution of variables. Continuous variables were expressed as mean ± standard deviation and compared with an independent-sample t-test. Categorical variables were expressed as numbers (%) and compared with the chi-square test. Repeated variables were compared with a one-way analysis of variance. Post hoc comparisons among the repeated variables in each group were performed by Bonferroni, if appropriate. A P-value of <0.05 was considered statistically significant.

## Results

Forty patients in the low-flow desflurane anesthesia group and 40 patients in the normal-flow desflurane anesthesia group were evaluated in this study. The baseline characteristics and laboratory parameters of the study groups are demonstrated in Table 1. Age, gender, body mass index, ASA status, and baseline laboratory parameters were similar between the two groups (Table 1).

**Table 1:** Comparison of the baseline characteristics of normal-flow and low-flow desflurane anesthesia ASA: American Society of Anesthesiologists.

	Normal-flow anesthesia (n = 40)	Low-flow anesthesia (n = 40)	P-value
Age, years	26.6 ± 7.7	25.8 ± 6.7	0.621
Gender, male (%)	14 (35)	20 (50)	0.175
Body mass index, kg/m^2^	22.2 ± 3.7	22.7 ± 3.4	0.565
ASA status			0.217
ASA I	26 (65)	31 (77.5)	
ASA II	14 (35)	9 (22.5)	
Duration of the procedure, min	114.5 ± 45.5	117.0 ± 27.6	0.770
Glucose, mg/dL	92.3 ± 11.7	92.8 ± 9.7	0.859
Urea, mg/dL	25.6 ± 8.1	25.4 ± 6.2	0.915
Creatinine, mg/dL	0.7 ± 0.2	0.7 ± 0.1	0.593
Sodium, mEq/L	138.9 ± 1.7	139.3 ± 1.4	0.283
Potassium, mEq/L	4.2 ± 0.2	4.3 ± 0.4	0.129
Hemoglobin, g/dL	13.6 ± 1.3	14.2 ± 1.5	0.067
Platelet, x10^3^/µL	253.2 ± 65.1	261.7 ± 54.9	0.548

**Figure 2:**
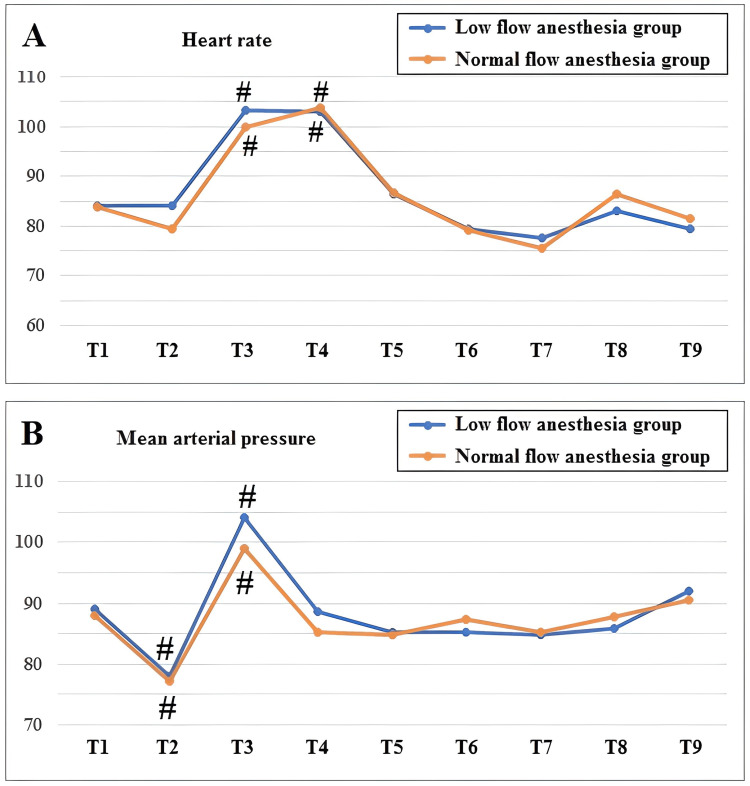
Comparison of heart rate (A) and mean arterial pressure (B) between two groups. T1: preoperative (basal), T2: immediately after anesthesia induction, T3: immediately after endotracheal intubation, T4: 5 min after endotracheal intubation, T5: 15 min after endotracheal intubation, T6: 30 min after endotracheal intubation, T7: 60 min after endotracheal intubation, T8: end of operation, T9: 15 min after the end of operation. #P < 0.05 when compared to the preoperative (T1) value within the same group.

MAP and HR values of the patients at each time point during the procedure are shown in Figure 2A, 2B. HR was significantly increased after the endotracheal intubation (T3) and 5 min after the endotracheal intubation (T4) compared to the preoperative values (T1) in both groups (Figure 2A). Also, MAP significantly decreased after the anesthesia induction (T2), whereas it increased after the endotracheal intubation (T3) when compared to the preoperative values (T1) in both groups (Figure 2B). However, there was no significant difference between the two groups in terms of HR and MAP when comparing the values at the same time points.

**Figure 3:**
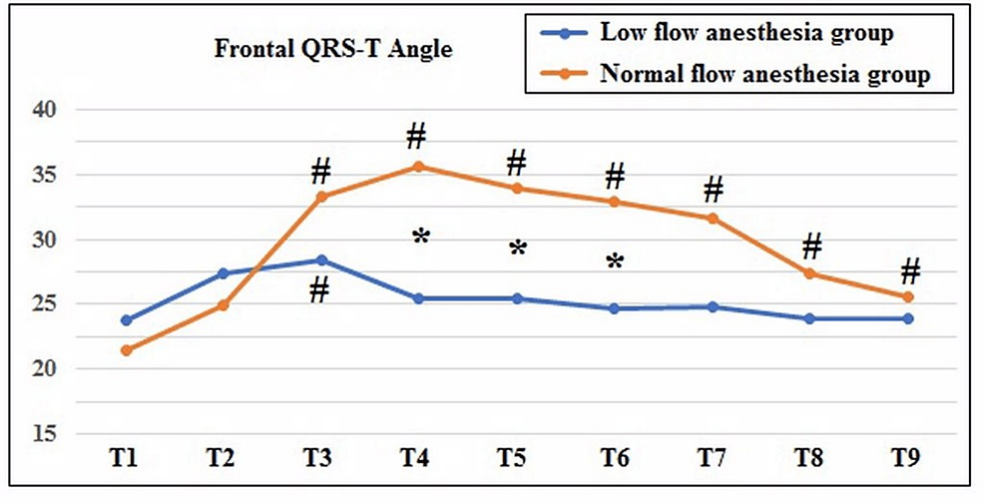
Comparison of frontal QRS-T angle between two groups. T1: preoperative (basal), T2: immediately after anesthesia induction, T3: immediately after endotracheal intubation, T4: 5 min after endotracheal intubation, T5: 15 min after endotracheal intubation, T6: 30 min after endotracheal intubation, T7: 60 min after endotracheal intubation, T8: end of operation, T9: 15 min after the end of operation. *P < 0.05 for the difference between the two groups at the same time point. #P < 0.05 when compared to the preoperative (T1) value within the same group.

Frontal QRS-T angles of the patients at each time point during the procedure are shown in Table 2 and Figure 3. In the LFA group, f(QRS-T)a increased after anesthesia induction, but this change did not reach significance when compared to the preoperative value (from 23.7 ± 12.9 to 27.4 ± 13.4, P = 0.119). However, it was significantly increased after endotracheal intubation when compared to the preoperative value (from 23.7 ± 12.9 to 28.5 ± 12.8, P = 0.003). No significant increase was observed at other time points compared to the preoperative value in the LFA group. In the NFA group, f(QRS-T)a was significantly higher at T3, T4, T5, T6, T7, T8, and T9 time points compared to T1 (Figure 3). On the other hand, f(QRS-T)a was significantly higher in the NFA group than in the LFA group at T4, T5, and T6 time points (Table 2).

**Table 2:** Comparison of frontal QRS-T angle between normal-flow and low-flow desflurane anesthesia during the procedure.

	Normal-flow anesthesia (n = 40)	Low-flow anesthesia (n = 40)	P-value
Preoperative, T1 (^o^)	21.5 ± 16.5	23.7 ± 12.9	0.494
Immediately after induction, T2 (^o^)	24.9 ± 17.0	27.4 ± 13.4	0.463
Immediately after intubation, T3 (^o^)	33.3 ± 22.0	28.5 ± 12.8	0.229
5 min after intubation, T4 (^o^)	35.6 ± 24.2	25.5 ± 12.2	0.022
15 min after intubation, T5 (^o^)	33.9 ± 1.3	25.5 ± 13.1	0.038
30 min after intubation, T6 (^o^)	32.9 ± 21.4	24.7 ± 12.6	0.042
60 min after intubation, T7 (^o^)	31.7 ± 20.2	24.8 ± 12.2	0.070
End of operation, T8 (^o^)	27.4 ± 18.6	23.9 ± 12.4	0.322
15 min after operation, T9 (^o^)	25.5 ± 18.0	23.9 ± 12.1	0.637

## Discussion

In this study, we aimed to compare the effect of LFA and NFA with desflurane on f(QRS-T)a. The main findings of our study were as follows: (I) although normal-flow desflurane anesthesia significantly increased the f(QRS-T)a during the procedure and this increase continued at 15 min after the postoperative period, low-flow desflurane anesthesia did not significantly increase the f(QRS-T)a except for immediately after endotracheal intubation, (II) f(QRS-T)a was significantly higher in the normal-flow desflurane anesthesia group compared to the low-flow desflurane anesthesia group at T4, T5, and T6 time points. To our knowledge, this is the first study reporting the protective effect of low-flow desflurane anesthesia on f(QRS-T)a.

Myocardial repolarization has a crucial role in the cardiac cycle, which plays a principal role in the development of arrhythmias. It has been traditionally calculated with QT and Tp-e interval measurements [17]. However, the calculation of QT and Tp-e interval measurements is difficult and requires software programs or additional tools such as a ruler and magnifying glass. Also, QT and Tp-e interval measurements are susceptible to noise and have poor reproducibility [18,19]. Therefore, researchers have focused on novel electrocardiographic markers, which are easily calculated from the surface ECG with high reproducibility. Frontal QRS-T angle is a novel marker of myocardial depolarization and repolarization heterogeneity and is defined as the absolute difference between myocardial depolarization and repolarization [10]. It can easily be measured from the automatic report of a 12-lead ECG. In addition, it was demonstrated that f(QRS-T)a was more accurate and reproducible, and less susceptible to noise than QT interval [9,20]. We, therefore, used f(QRS-T)a in this study for the evaluation of the myocardial depolarization and repolarization heterogeneity.

Endotracheal intubation, anesthetic agents, and the type of anesthesia may affect myocardial repolarization. Previous studies demonstrated that endotracheal intubation itself prolonged myocardial repolarization [16,21]. Similarly, we also detected in our study that f(QRS-T)a was significantly increased immediately after the endotracheal intubation both in the LFA and NFA groups when compared to the preoperative values. On the other hand, there was no significant difference between the LFA and NFA groups in terms of f(QRS-T)a after the endotracheal intubation. These results suggest that endotracheal intubation prolongs the myocardial repolarization independent of the anesthesia type. The main reason for this prolongation is the sympathetic activation occurring due to the catecholamine discharge during endotracheal intubation [22].

The effect of volatile anesthetic agents on myocardial repolarization parameters has been investigated in previous studies [23]. It was demonstrated that desflurane anesthesia prolonged QT interval [6,7,13,14]. However, to our knowledge, no study has evaluated the effect of low-flow desflurane anesthesia and normal-flow desflurane anesthesia on the f(QRS-T)a. In our study, we found that NFA significantly increased the f(QRS-T)a during the procedure and this increase continued at 15 min after the postoperative period, whereas LFA did not significantly increase the f(QRS-T)a except for immediately after endotracheal intubation. In addition, we detected that this increase in f(QRS-T)a was significantly higher in the NFA compared to the LFA group. Our results indicate that NFA increases f(QRS-T)a more than the LFA does. Therefore, it may be suggested that NFA may have a higher arrhythmogenic effect than LFA. However, we did not observe any clinical arrhythmia during the study period. This may be because we included low-risk patients without any known cardiac disease. We think that further prospective studies with a larger group of participants are required to better elucidate the effect of different gas flow rates on myocardial repolarization parameters in different patient populations (high-risk patients and/or known cardiac disease).

The effect of LFA on hemodynamic parameters was evaluated in many studies [3,24-26]. It was found that LFA did not significantly change the hemodynamic parameters when compared to NFA. We also evaluated the hemodynamic parameters including HR and MAP in this study. Similar to these studies, we found no intra-group differences in terms of HR and MAP at any time point. Our results support that the LFA method provides effective stabilization without any hemodynamic disturbance and can be used safely.

Our study had some limitations. First, the sample size was relatively small. Second, anesthesia maintenance was provided according to the MAC, and it might be more suitable to maintain the anesthesia according to the MACage. However, we excluded the patients under the age of 18 and over the age of 65 in this study. Therefore, we do not think that using MAC instead of MACage affects our results. Third, 24-h Holter ECG recording was not performed. It would provide additional contribution to perform 24-h Holter ECG recording and evaluating the effect of different gas flow rates on arrhythmias. However, we did not observe any clinical arrhythmia during the hospital stay. Fourth, we evaluated the low-risk patients (ASA I and II) without any cardiac disease in this study. Therefore, our results cannot be generalized to all patient groups.

## Conclusions

Frontal QRS-T angle is a novel marker of myocardial depolarization and repolarization heterogeneity. In this study, we found that NFA significantly increased f(QRS-T)a, whereas LFA did not significantly increase the f(QRS-T)a except for immediately after endotracheal intubation. It was also detected that f(QRS-T)a was significantly higher in the NFA group compared to the LFA group. Therefore, it can be concluded that LFA has more protective effects on myocardial repolarization than NFA.
